# Rhesus macaques compensate for reproductive delay following ecological adversity early in life

**DOI:** 10.1002/ece3.8456

**Published:** 2022-01-12

**Authors:** Logan Luevano, Chris Sutherland, Stephanie J. Gonzalez, Raisa Hernández‐Pacheco

**Affiliations:** ^1^ Department of Biological Sciences California State University‐Long Beach Long Beach California USA; ^2^ The Center for Research into Ecological and Environmental Modeling University of St. Andrews St. Andrews UK

**Keywords:** Cayo Santiago, density dependence, fitness, hurricanes, PAR

## Abstract

Adversity early in life can shape the reproductive potential of individuals through negative effects on health and life span. However, long‐lived populations with multiple reproductive events may present alternative life history strategies to optimize reproductive schedules and compensate for shorter life spans. Here, we quantify the effects of major hurricanes and density dependence as sources of early‐life ecological adversity on Cayo Santiago rhesus macaque female reproduction and decompose their effects onto the mean age‐specific fertility, reproductive pace, and lifetime reproductive success (LRS). Females experiencing major hurricanes exhibit a delayed reproductive debut but maintain the pace of reproduction past debut and show a higher mean fertility during prime reproductive ages, relative to unaffected females. Increasing density at birth is associated to a decrease in mean fertility and reproductive pace, but such association is absent at intermediate densities. When combined, our study reveals that hurricanes early in life predict a delay‐overshoot pattern in mean age‐specific fertility that supports the maintenance of LRS. In contrast to predictive adaptive response models of accelerated reproduction, this long‐lived population presents a novel reproductive strategy where females who experience major natural disasters early in life ultimately overcome their initial reproductive penalty with no major negative fitness outcomes. Density presents a more complex relation with reproduction that suggests females experiencing a population regulated at intermediate densities early in life will escape density dependence and show optimized reproductive schedules. Our results support hypotheses about life history trade‐offs in which adversity‐affected females ensure their future reproductive potential by allocating more energy to growth or maintenance processes at younger adult ages.

## Graphical abstract

The Cayo Santiago rhesus macaque population presents a novel reproductive strategy where females experiencing major natural disasters early in life overcome their initial reproductive penalty and exhibit no major negative fitness outcomes. Such strategy suggests that investing more energy into development and maintenance at younger ages allows long‐lived females experiencing early‐life ecological adversity to reproduce at a mean rate equivalent to that of a typical female cohort later in life. Density at birth presents a more complex relation with reproduction that suggests females experiencing a population regulated at intermediate densities early in life escape density‐dependent effects in reproduction.

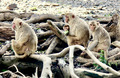

## 1 INTRODUCTION

Adversity early in life can have negative effects on the reproductive performance of individuals, and thus influences life history evolution and population fitness (Douhard et al., 2014; Lindström, 1999; Lu et al., 2019; Nussey et al., 2007). For example, nutritional adversity early in life (e.g., competing siblings and dietary restrictions) is associated with shorter life spans in rats (Desai & Hales, 1997), birds (Lindström, 1999), and primates (Campos et al., 2021; Tung et al., 2016), as well as a delay in reproductive debut (Nussey et al., 2007) and consequent lower reproductive success in ungulates (Douhard et al., 2014; Rose et al., 1998). Such predisposition to hardship later in life can be explained through cohort effects in which observed differences in health and consequent fitness components are shared among individuals subjected to the same environmental conditions during developmental stages, such as adversities experienced in utero and exposure to environmental hazards during immature stages (Gaillard et al., 2003; Garrott et al., 2012; Lindström, 1999; Lindström & Kokko, 2002; O'Rand, 1996; Payo‐Payo et al., 2016). As evidence supporting early‐life adversity and cohort effects frameworks continue emerging, it is crucial to understand demographic mechanisms for life history optimization following adverse conditions early in life.

A potential adaptive strategy for individuals exposed to early‐life adversity involves changes in the pace of reproduction due to predicted shorter life spans (e.g., predictive adaptive response model [PAR]; Bateson et al., 2014; Gluckman et al., 2005; Nettle et al., 2013). In such a scenario, selection is hypothesized to optimize the reproductive schedule of individuals who experience adversity during developmental stages by accelerating reproduction to maintain a higher lifetime reproductive success (LRS; Belsky et al., 1991; Draper & Harpending, 1982). Consistent with this, adversity early in life has been associated with younger ages of reproductive debut among many mammals (Douhard et al., 2014; Mumby et al., 2015; Sloboda et al., 2009), including humans (Belsky, 2019; Nettle et al., 2011; Rickard et al., 2014). However, recent evidence suggests that accelerated reproduction may not be an adaptive response to early‐life adversity in nonhuman primates as early‐life adversity in female baboons did not accelerate their reproduction, and thus was not associated with high LRS maintenance (Weibel et al., 2020). These findings open questions regarding other potential evolutionary strategies that long‐lived populations develop to cope with early‐life adversity and compensate for shorter life spans. Because life history theory also predicts fitness costs as a result of accelerated reproduction (e.g., reduced size and suppressed fecundity; Stearns, 1992), these recent findings on long‐lived animals may not be isolated (Snyder‐Mackler et al., 2020).

In this study, we quantify the effects of early‐life ecological adversity in a long‐living nonhuman primate and evaluate potential demographic mechanisms for optimal reproductive success later in life. We focus on major hurricanes and density dependence as main sources of ecological adversity on the rhesus macaque population at Cayo Santiago. Cayo Santiago, located in the Caribbean region, is subjected to major hurricane events which suppress mean annual fertility (Morcillo et al., 2020) and change the social structure of the population (Testard et al., 2021). Moreover, this population is known to be regulated by density through negative density dependence in fertility (Hernández‐Pacheco, Delgado, et al., 2016; Hernández‐Pacheco et al., 2013). Apart from being a nutritional adversity, density dependence at Cayo Santiago is likely associated with increased prevalence of aggressive interactions resulting in the suppression of female fertility (Dettmer et al., 2014; Judge & De Waal, 1997; Sterck et al., 1997). Thus, major hurricane events and increased population density early in the life of Cayo Santiago rhesus macaque females may represent sources of both nutritional and psychosocial adversities.

In contrast to the PAR hypothesis of accelerated reproduction, we hypothesize that early‐life adversity suppresses reproduction at younger adult ages and evaluate alternative demographic mechanisms for the maintenance of LRS. First, to test whether adversity early in life reduces mean fertility in particular age groups, we investigate variation in mean age‐specific fertility. In such a scenario, a life history strategy for optimal reproductive schedules may involve the increased reproductive performance of females during prime reproductive ages. Second, we quantify the effects of early‐life ecological adversity on reproductive pace (i.e., reproductive debut and birth skipping). Adversity may decrease the reproductive pace of females by delaying reproductive debut. Here, optimizing reproductive success later in life may involve a lower frequency of birth skipping. Finally, we quantify whether females experiencing harsh environments early in life attain a different LRS relative to females not experiencing adversities. No reduction in LRS among females who experience adversity would suggest the evolution of demographic mechanisms for optimal reproductive success later in life.

## 2 METHODS

### 2.1 Study population

**FIGURE 1:**
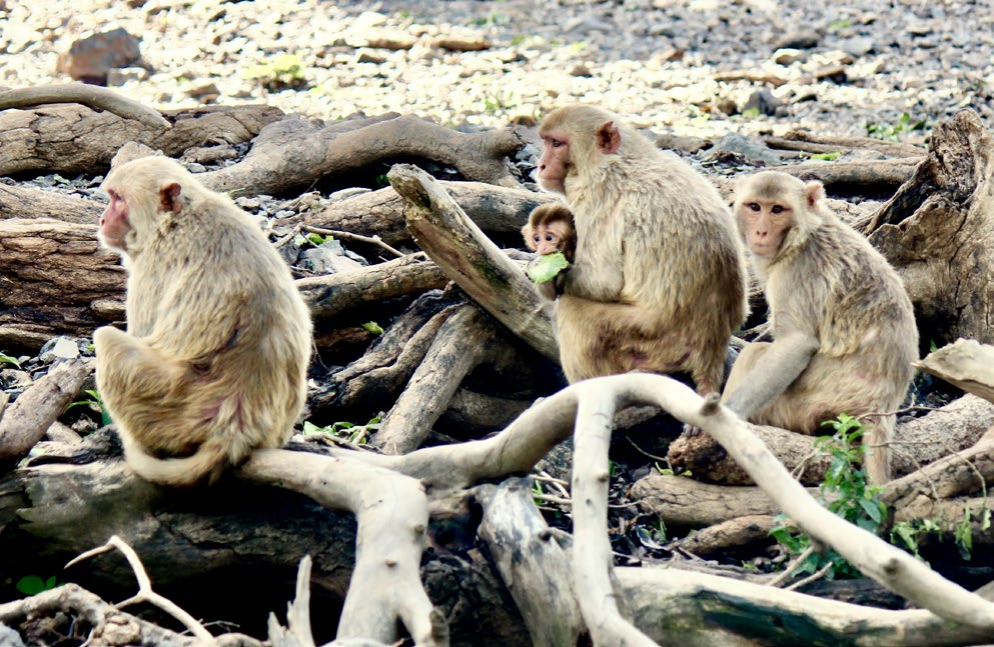
Cayo Santiago rhesus macaque females with an infant

The Cayo Santiago Field Station (CSFS) is a 15.2‐ha island that serves as a research facility managed by the Caribbean Primate Research Center of the University of Puerto Rico for behavioral and noninvasive research. Located 1 km off the southeastern coast of Puerto Rico (18°09′N, 65°44′W), the CSFS is inhabited by a population of free‐ranging rhesus macaques (*Macaca mulatta*), all of which descended from a group of 409 individuals released onto Cayo Santiago in 1938. Since establishment, the population has been maintained under seminatural conditions allowing for the natural formation of social groups, social rank, and mating seasons (Rawlins & Kessler, 1985; Figure 1). Monkeys forage on vegetation, spending 50% of their time on average eating vegetation found on the island (Marriott et al., 1989). The population is also provisioned with ad libitum, high‐protein monkey chow rationed at approximately 0.23 kg/monkey/day and ad libitum drinking water via automatic drinkers located throughout the island. Veterinary intervention is restricted to the annual trapping season in which yearlings are trapped, marked for identification using ear notches and a unique ID tattoo, physical samples are collected, and tetanus inoculation at 1 year of age and booster at 2 years of age are administered. During trapping, some individuals are permanently removed from the island to control for population size. Several management practices have taken place annually (i.e., no removal, removal of entire social groups, and removal of specific age classes) that, together with natural deaths, have resulted in significant changes in density through the history of the population (range: ~400 to 1700 individuals alive in the population; see Hernández‐Pacheco, Delgado, et al., 2016). Since 1956, the population has been monitored through visual censuses resulting in a demographic database which includes the date of birth, sex, matrilineage, and date of death or permanent removal of the population for all individuals. Births, deaths, and removals are reported within 2 days of occurrence (Ruiz‐Lambides et al., 2017). During data collection, all applicable institutional and/or national guidelines for the care and use of animals were followed.

### 2.2 *Early*‐*life ecological adversity*

We addressed the effects of two ecological sources of early‐life adversity on reproduction: major hurricanes and population density. We defined early life as the period from gestation (i.e., in utero) to immaturity (<3 years of age). The gestation period was estimated by subtracting 165 days from the date of birth for each individual (Rawlins & Kessler, 1985). Since the establishment of census records, the CSFS has experienced the direct effect of three major hurricanes (Category ≥ 3): Hugo (September 18, 1989), Georges (September 21, 1998), and Maria (September 20. 2017; Morcillo et al., 2020). We focused only on hurricanes Hugo and Georges as individuals experiencing Maria did not have complete reproductive life histories at the moment of our study. Hurricanes Hugo and Georges were category 3 hurricanes when their centers were closest to the CSFS (~23 and ~8.4 km from CSFS, respectively). These hurricanes exhibited sustained wind speeds of approximately 201 and 185 km h^−1^, respectively, with hurricane‐force winds extending over the entire field station (Morcillo et al., 2020). Both hurricanes produced severe damage to Cayo Santiago's vegetation, with 60–90% canopy loss following each hurricane (Morcillo et al., 2020). Although food provisioning, and thus census taking, resumed after 1 (Hugo) and 2 (Georges) days (Morcillo et al., 2020), major hurricanes are significant events that also cause changes in behavior involving an increase in the proximity of social networks (Testard et al., 2021). Given negative density dependence in reproduction driven by the annual number of adult females alive in the population (Hernández‐Pacheco et al., 2013; Hernández‐Pacheco & Steiner, 2017), we also considered the experienced female adult density at birth as a second source of early‐life ecological adversity. For this, we defined population density as the total number of adult females (≥3 years of age) alive at the onset of each birth season. Cayo Santiago monkeys exhibit reproductive synchrony with 73% of births occurring in a 3‐month period (Hernández‐Pacheco, Rawlins, et al., 2016). Density at the onset of the birth season (range: 72–468 adult females) represents more accurately the experienced density early in life as opposed to the density at birth for a particular female due to the potential variation in density caused by culling events, especially late in the birth season. Although density at the onset of the mating season may also be an important predictor of late‐life reproduction, our population does not exhibit large differences in adult female density 5.5 months prior to the onset of the birth season as no significant deaths or culling occurs. With this information, all females were classified into two cohort types: a hurricane cohort and a nonhurricane cohort, each with a corresponding experienced female adult density at the onset of the birth season. The hurricane cohort included all female adults that experienced hurricanes Hugo or Georges, either in utero or as immatures, and the nonhurricane cohort included all females born during the study period that did not experience these hurricanes early in life.

### 2.3 Statistical analysis of reproductive metrics

To quantify the effects of early‐life ecological adversity on reproduction, we used four different metrics to characterize fertility, pace of reproduction, and reproductive output. We addressed fertility across the life span by measuring mean age‐specific fertility, the pace of reproduction by measuring age at reproductive debut and age‐specific birth skipping probability past debut, and reproductive output by measuring LRS. Our analysis included all females born in the 43‐year period between 1973 and 2016 that survived into adulthood (3 years of age) for a total of 2108 reproductive histories (*n*_hurricane_ = 423 females; *n*_nonhurricane_ = 1685 females). Females were monitored until December 2020. Females culled or still alive in the population at the end of the monitoring period were right censored. For all reproductive metrics, we considered all live births born to each female, and thus attributed offspring fitness directly to the offspring, not mothers (Weibel et al., 2020). In this way, we avoid combining maternal and offspring phenotypes as the phenotype of the offspring already combines both direct and maternal selection (Wolf & Wade, 2001).

To address the effects of early‐life adversity on the mean age‐specific fertility rate, we tracked each adult female over time and recorded whether she produced an offspring or not at each age. We analyzed these data using generalized additive mixed models (GAMMs). These models are nonparametric extensions of generalized linear models that allow the evaluation of nonlinear relationships and thus are appropriate to model age‐specific reproductive metrics in primates. For this, we used a logit link function for the binary outcome of whether a female reproduced in a given year. We considered cohort type (born in a hurricane or nonhurricane year) as a fixed effect and age and density as smooth terms. We fitted a series of competing models that included cohort as both an additive effect, that is, intercepts vary but smooth terms are consistent across cohorts, and as an interactive effect, that is, intercepts and the smooth terms vary by cohort. We also included a random intercept of individual ID to account for unobserved traits of our focal females and repeated measurements. Considering all factor combinations resulted in a total of nine competing models that were evaluated using the Corrected Akaike's Information Criterion (AIC_c_) to address potential overfitting (Burnham & Anderson, 2002). All models were run in R version 4.0.3 (R Core Team, 2020) using the gamm4 package (Wood & Scheipl, 2020).

To address the effects of early‐life adversity on reproductive pace, we estimated the age at reproductive debut and the frequency of birth skipping across the reproductive life span of females past debut. We measured reproductive debut as the age at which females gave birth to their first live offspring. Given the reproductive synchrony of the population, age at delivery exhibits a multimodal distribution described by pulses within age classes, and thus, attaining normality in the data is not possible. Because of this, we followed Pittet et al. (2017) and binned the data into discrete age classes of reproductive debut of 3, 4, or 5 years of age and tested whether cohort type or increasing density predicted age at reproductive debut using ordinal logistic regression models evaluated with AIC_c_. We checked that the proportional odds assumption held (*p* = .54; see Appendix S1). We determined birth skipping by tracking each female over time past their reproductive debut and recording whether she skipped a birth season or not across the remaining ages of her reproductive life. For this, we used a logit link function for the binary outcome of whether a female reproduced (nonskipper) or not (skipper) in a given year and analyzed the data using GAMMs. We considered cohort type as a fixed effect and age and density as smooth terms. Competing models included cohort as both an additive and an interactive effect. We also included a random intercept of individual ID to account for unobserved traits of our focal females and repeated measurements. We fitted nine competing models and evaluated them using AIC_c_.

To address the effects of early‐life adversity on reproductive output, we estimated LRS as the total number of live offspring born to each female. To avoid biasing our analysis to females with short life spans, for this analysis, we truncated our sample size to data from 1973 to 1996 as these female birth cohorts potentially experienced their entire reproductive life span in Cayo Santiago by the end of the monitoring period (3–24 years of age; *n*_(hurricane)_ = 102 and *n*_(nonhurricane)_ = 288). Females removed from the population were not included in the analysis. To test the influence of hurricanes and density on LRS and given the right skewed distribution of LRS, we employed zero‐inflated regression models with negative binomial distribution evaluated with AIC_c_. Prior to running the models, we determined the data followed a negative binomial error distribution by testing for overdispersion in the count portion of the model using the AER package in R (Kleiber & Zeileis, 2020; dispersion test: *c* = 1.29, *z* = 3.44, *p* < .001) and were zero inflated by comparing model fit with the nonzero‐inflated analog using the pscl package (Jackman, 2020) in R. As life span is known to be a critical factor in determining LRS of long‐lived primates (Blomquist, 2013; Robbins et al., 2011; Weibel et al., 2020), we controlled for age at death in both the count and the inflated part of our models. First, we tested the effects of cohort type and density in the count model. As there was no support for cohort effects, we did not include this covariate in the count model when evaluating the final list of competing models. We fitted a final list of four models that included density as covariate of the count model and all combinations of density and cohort type in the zero‐inflated parts of the model.

Finally, to explore whether the studied ecological early‐life adversities are associated to shorter life spans, we quantified the contribution of major hurricanes and density at birth early in life to mortality pressure. For this, we analyzed survival using a Cox proportional hazards model that included cohort type and density as fixed effects. We analyzed survival of all females born during the entire study period (1973–2016), regardless of their time of death (*n* = 4236). Those culled or alive at the end of the study period were right censored. Because the proportional hazards assumption was not met for the density variable, suggesting that the association between density early in life and mortality changes over time (Appendix S2), we extended our model using time‐varying coefficients by stratifying density into four age periods following visual inspection of the estimated coefficient for density across time (Lee et al., 2020; Zhang et al., 2018; Appendix S3): birth to 0.02 years (approximately first week of life), 0.02–3.5 years, 3.5–16.5 years, and older than 16.5 years of age. The resulting stratified Cox model satisfied the proportional hazards assumption (*p* = .15; Appendix S4).

## 3 RESULTS

**FIGURE 2:**
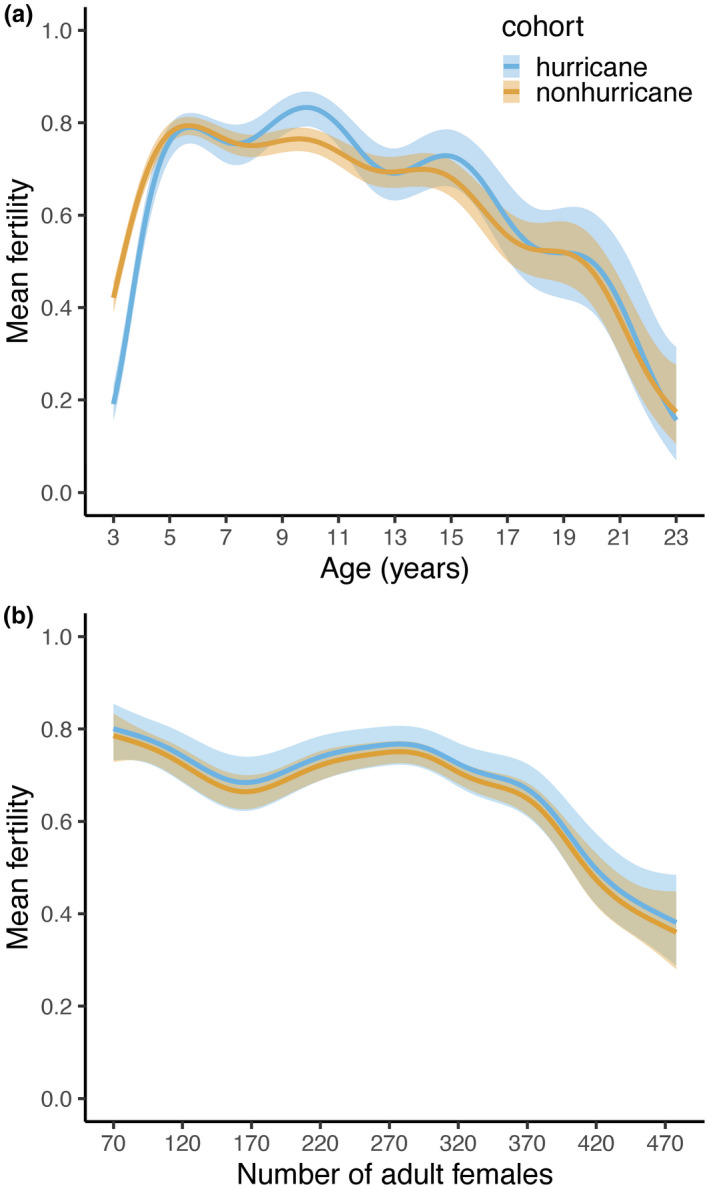
Model predictions for (a) mean age‐specific fertility of female rhesus macaques experiencing a major hurricane early in life (blue), relative to unaffected females (orange), holding density at average value, and (b) mean fertility of females experiencing increasing density early in life, holding age at average value. Ribbons represent 95% confidence intervals

Mean age‐specific fertility was found to be driven by both major hurricanes and density at birth (*n* = 14,401, *adjR* ^2^ = .12; cumulative model weight = 0.90; Figure 2a; Table 1; Appendix S5). The model included a varying intercept for cohort type, a factor smooth interaction between age and cohort type and a smooth term for density (Table 1; Appendix S6). The hurricane cohort had an overall lower mean fertility than the nonhurricane cohort ($\beta_{\mathrm{nonhurricane}}\pm SE=\;0.157\pm 0.060$), but mean fertility across cohort type was dependent on age. The data show that females in the hurricane cohort exhibited a lower probability of successfully reproducing during early ages (3–4 years; 0.357 ± 0.479, mean ± *SD*), relative to the nonhurricane cohort females (0.474 ± 0.499; Figure 2a). However, females in the hurricane cohort had a higher probability of successfully reproducing during typical prime reproductive ages for this population (5–16 years; 0.733 ± 0.442; Hernández‐Pacheco et al., 2013), relative to females in the nonhurricane cohort (0.702 ± 0.457). Density showed a nonlinear association with mean fertility. As density at birth increased, mean fertility decreased except at intermediate densities (~170–300 adult females; Figure 2b; Table 1).

**TABLE 1:** Coefficients of the top model describing mean age‐specific fertility of Cayo Santiago rhesus macaque females as a function of early‐life ecological adversity, cohort type, and population density, with individual ID as random intercepts

Parameter	Estimate	*SE*
$\beta_{\left(\mathrm{intercept}\right)}$	0.381	0.054
$\beta_{\left(\mathrm{nonhurricane}\right)}$	0.157	0.060

Smooth terms	Estimated degree of freedom	
s(age):hurricane	8.097	
s(age):nonhurricane	8.172	
s(density)	7.041	

Note

“:” stands for factor smooth interaction.

**FIGURE 3:**
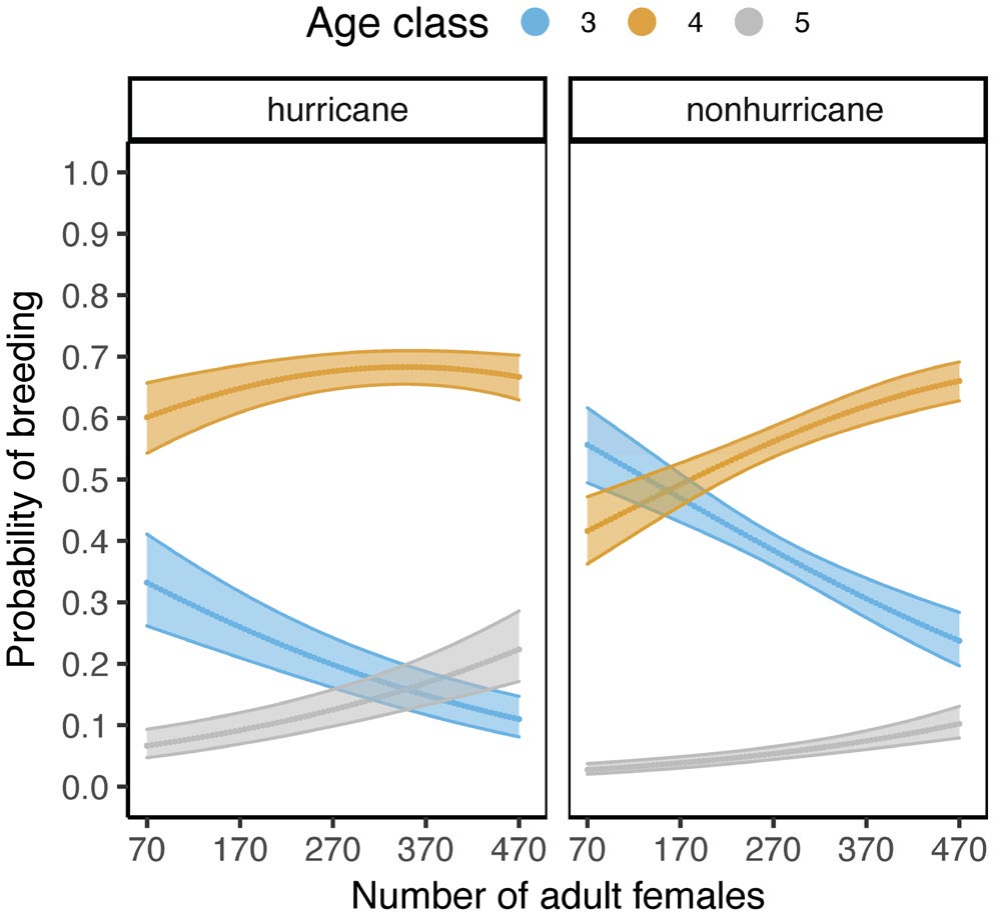
Predicted joint effect of major hurricanes and increasing density early in life on age at reproductive debut of female rhesus macaques. Blue represents age class 3; orange represents age class 4; and gray represents age class 5. Ribbons represent 95% confidence intervals

**FIGURE 4:**
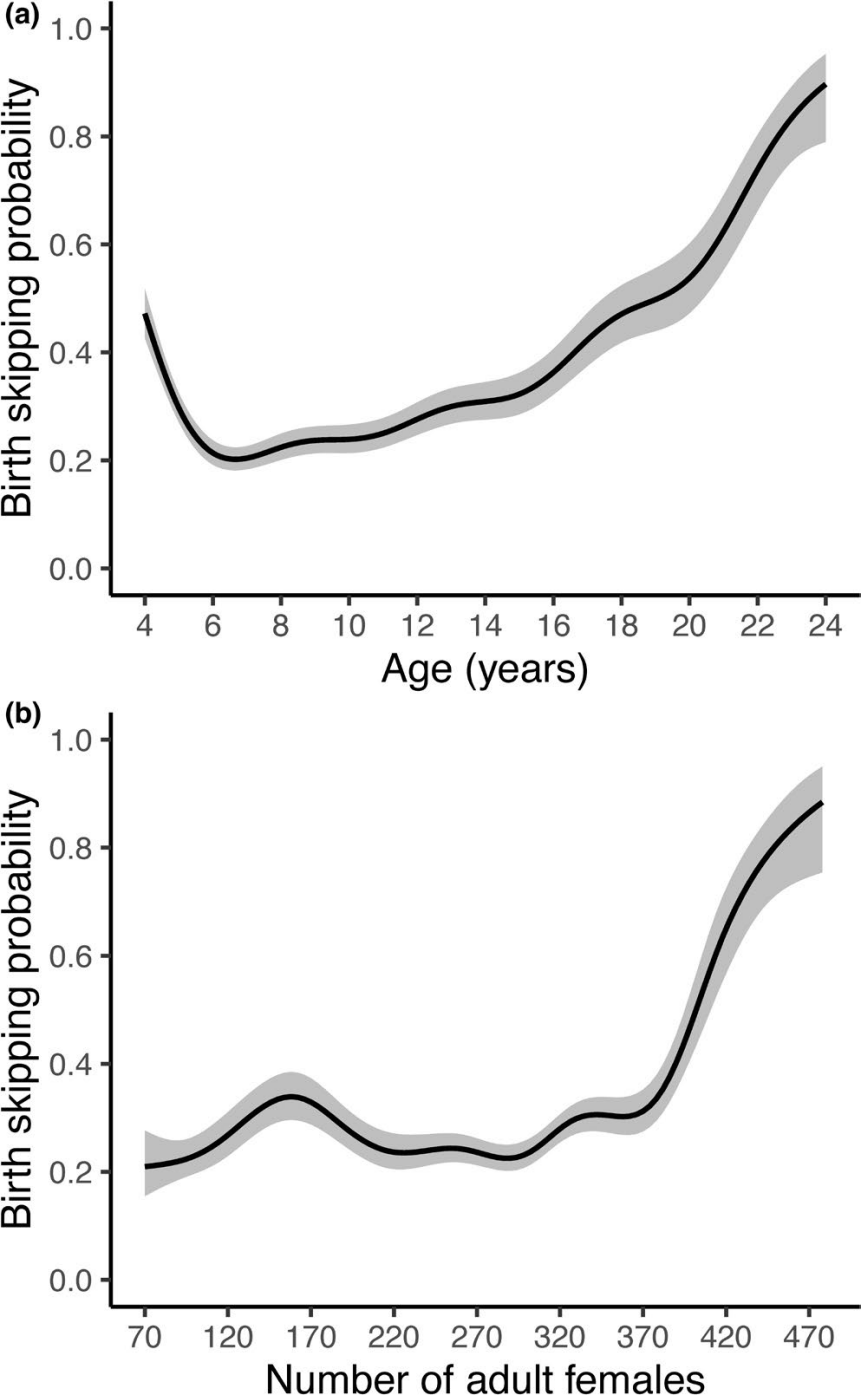
Model predictions for (a) mean age‐specific birth skipping probability past reproductive debut of female rhesus macaques holding density at average value and (b) birth skipping probability past reproductive debut across density holding age at average value. Ribbons represent 95% confidence intervals

Age at reproductive debut varied from 3.003 to 8.953 years of age. Females experiencing a major hurricane early in life showed a delayed sexual maturity, debuting at a median age of 4.690 years (95% CI: 3.885, 6.002) in contrast to females from the nonhurricane cohort who debuted at a median age of 4.074 years (95% CI: 3.724, 5.790). The model indicated that females in the hurricane cohort were more likely to give birth at later ages (relative to age class 3) 2.52 times that of females in the nonhurricane cohort, holding constant all other variables ($\beta_{\mathrm{nonhurricane}}\pm SE=\;‐0.925\pm 0.138$; *n* = 1653; cumulative model weight = 1.0; Figure 3; Appendices S7 and S8). For every one unit increase in density, the odds of being more likely to give birth at later ages increase by 0.3%, holding constant all other variables (0.003±0.001; Figure 3). The probability of skipping a birth season past reproductive debut was driven by age and density at birth (*n* = 10,740, *adjR* ^2^ = .06; cumulative model weight = 0.66; Table 2; Figure 4; Appendix S9). Age‐specific effects were nonlinear with very young and very old females exhibiting a higher probability of skipping the corresponding birth season, holding density at its mean value (Figure 4a). Increasing density increased the mean birth skipping probability when holding age at its mean value, especially at extreme values (<160 and >370 adult females; Table 2; Figure 4b).

**TABLE 2:** Coefficients of the top model describing the birth skipping probability of Cayo Santiago rhesus macaque females as a function of age and population density at birth with individual ID as random intercepts

Parameter	Estimate	*SE*
$\beta_{\left(\mathrm{intercept}\right)}$	−0.760	0.026

Smooth terms	Estimated degree of freedom	
s(age)	7.768	
s(density)	8.015	

**FIGURE 5:**
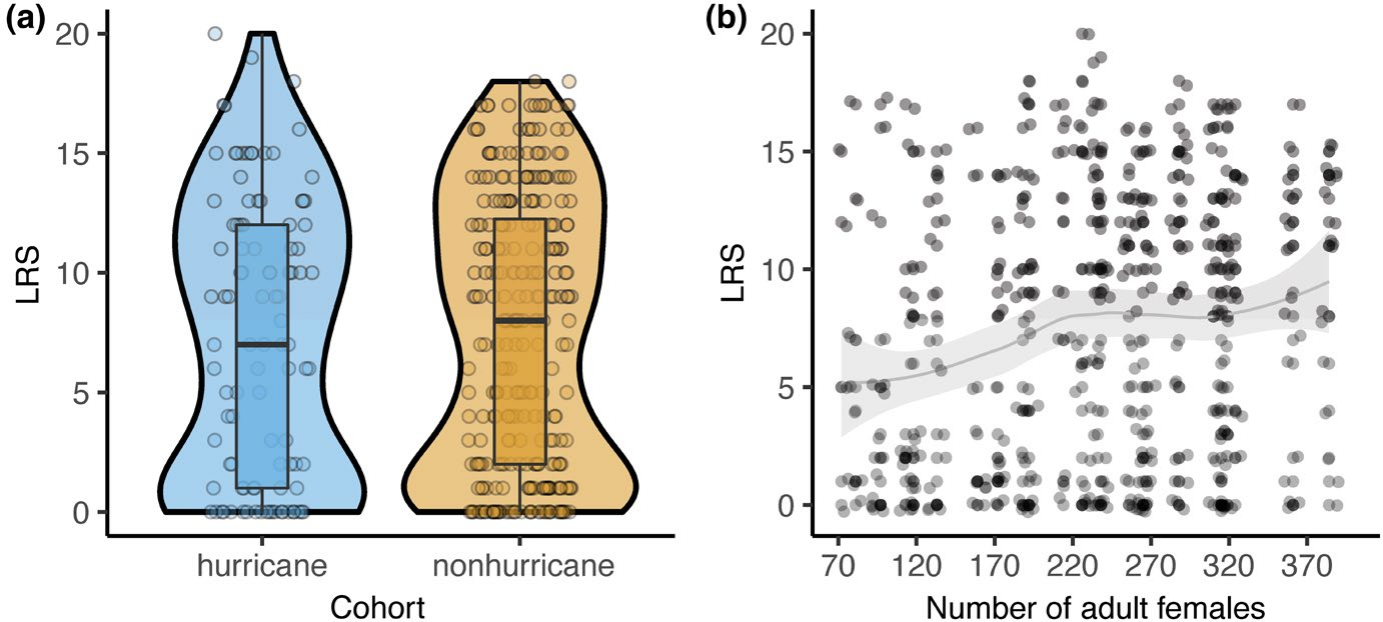
Lifetime reproductive success for female rhesus macaques (a) across cohort type and (b) density with a smoothed conditional mean for visual patterns. Blue, hurricane cohort; orange, nonhurricane cohort. These empirical data represent females living their entire reproductive history in Cayo Santiago (birth seasons from 1973 to 1996). Ribbons represent 95% confidence intervals

Among adult females with a chance of giving birth (count portion of the model), we found no cohort type effects on LRS, but LRS increased with increasing density and age at death (cumulative model weight = 0.65; Table 3; Appendix S10). Females in the hurricane cohort with LRS >0 had a median LRS of 10 offspring (95% CI: 1, 18; mean ±SE: 9.21 ± 0.56), whereas females in the nonhurricane cohort had a median LRS of nine offspring (95% CI: 1, 17; mean ± *SE*: 8.67 ± 0.33). However, the odds of being among those with LRS = 0 decreased by 0.0007 for females belonging to the nonhurricane cohort, relative to the baseline measure (β±SE=‐7.203±3.136; Table 3). When including all females, the median LRS for females in the hurricane and nonhurricane cohorts were seven offspring (95% CI: 0, 17.5; mean ± *SE*: 7.31 ± 0.58) and eight offspring (95% CI: 0, 17.0; mean ± *SE*: 7.41 ± 0.33), respectively (Figure 5a). For females with the chance of reproducing, LRS increased by 1.0007 with a one‐unit increase in density (β±SE=0.0007±0.0002; Table 3; Figure 5b). The odds of being among those with LRS = 0 also increased for females belonging to high‐density birth seasons (0.020±0.009; Table 3). A unit increase in age at death increased LRS by 1.090 times among those who had the chance of reproducing (0.089±0.003) and decreased the odds of having LRS = 0 by 0.00007 (−9.640±3.531; Table 3).

**TABLE 3:** Coefficients of the top model describing lifetime reproductive success of Cayo Santiago rhesus macaque females as a function of early‐life ecological adversity, cohort type and population density, and age at death

Parameter	Estimate	*SE*
*Count model*		
$\beta_{\left(\mathrm{intercept}\right)}$	0.472	0.081
$\beta_{\left(\mathrm{density}\right)}$	0.0007	0.0002
$\beta_{\left(\mathrm{age}\;\mathrm{at}\;\mathrm{death}\right)}$	0.089	0.003
*Zero‐inflation model*		
$\beta_{\left(\mathrm{intercept}\right)}$	42.642	16.004
$\beta_{\left(\mathrm{density}\right)}$	0.020	0.009
$\beta_{\left(\mathrm{nonhurricane}\right)}$	−7.203	3.136
$\beta_{\left(\mathrm{age}\;\mathrm{at}\;\mathrm{death}\right)}$	−9.640	3.531

**FIGURE 6:**
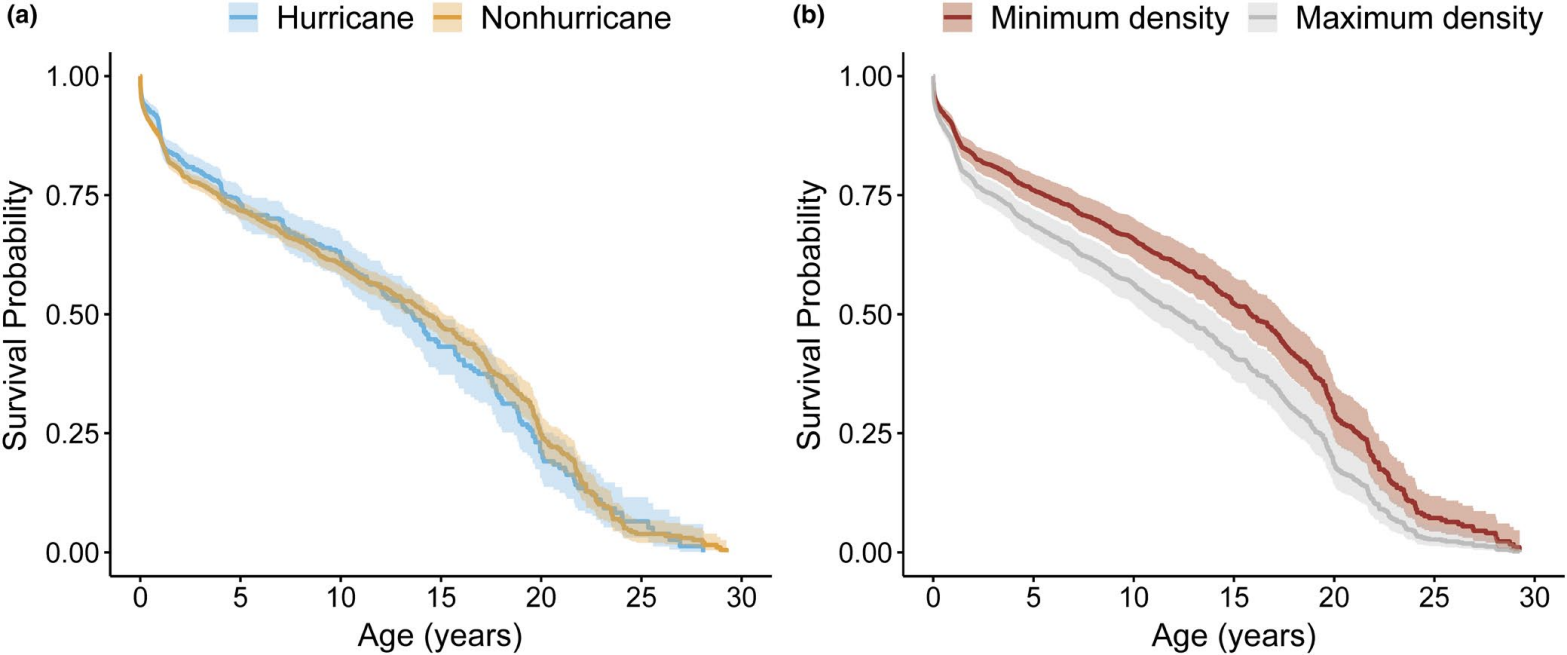
Kaplan–Meir curves for female rhesus macaques (a) across cohort type and (b) minimum (72 adult females) and maximum (468 adult females) density. Blue, hurricane cohort; orange, nonhurricane cohort; red, minimum density; gray, maximum density. Ribbons represent 95% confidence intervals

Major hurricanes early in life did not have a significant effect on late‐life survival (Figure 6a; Appendix S11). However, our analysis showed that density affects survival significantly but mostly at immature and old ages (cumulative model weight = 0.64; Figure 6b, Appendix S12). Out of the 4236 females, 120 females died during the first week of life (<0.02 years). At this early period, a one‐unit increase in density reduced the risk of death by 0.62% (hazard ratio = 0.994; CI: 0.992, 0.996; Figure 6b). However, all other age periods experienced an increase in the risk of death at each age with increasing density. For every one unit increase in density, risk of death increased by 0.02% for females between the ages of 0.02 and 3.5 years (total deaths =768; hazard ratio =1.002; 95% CI: 1.001, 1.003) and females of age >16.5 years (total deaths = 175; hazard ratio = 1.002; 95% CI: 1.000, 1.004; Figure 6b). In contrast, the survival of females between the ages of 3.5 and 16.5 years was not significantly affected by density (total deaths = 453).

## 4 DISCUSSION

Cayo Santiago rhesus macaque females exposed to major hurricanes early in life exhibit a delayed reproductive debut but maintain reproductive pace past debut and exhibit a higher mean fertility during prime reproductive ages resulting in no major differences in LRS relative to females experiencing no hurricanes. Such strategy suggests that investing more energy into development and maintenance at younger ages allows females experiencing early‐life ecological adversity to reproduce at a mean rate equivalent to that of typical females later in life. Increasing density early in life was associated to a lower mean fertility, a delayed reproductive debut, and a slower reproductive pace past debut, but most of these associations became weaker at intermediate densities. Our study reveals a nonlinear relationship between density and reproduction suggesting that females experiencing intermediate density values at birth can escape negative density‐dependent effects on reproduction.

### 4.1 Hurricane‐induced early‐life adversity

Two different reproductive life history strategies were observed between the hurricane cohort (i.e., adversity‐affected strategy) and the nonhurricane cohort (i.e., typical strategy). The adversity‐affected strategy involved delaying reproductive debut. Harsh conditions early in life, especially those involving nutritional and psychosocial adversities, may delay reproductive debut by imposing constraints on development (e.g., low body fat storage and low nutrient intake; Monaghan, 2008) due to associations between birth weight and behavioral and physical performance later in life (Zablocki‐Thomas et al., 2017). However, adverse‐affected females exhibited no difference in reproductive pace (i.e., probability of birth skipping) past debut relative to nonaffected females and showed a higher mean fertility during prime reproductive ages (i.e., 5–16 years of age). Later reproductive debut may be associated with faster postpartum recuperation (Mas‐Rivera & Bercovitch, 2008), potentially allowing early‐life adversity affected females to maintain their reproductive pace. Such strategy contributed to late‐life reproductive compensation among females experiencing a major hurricane early in life.

Delaying early reproduction and compensating for it later may support LRS maintenance among Cayo Santiago adult females experiencing major natural disasters early in life because of their high mean survival, long life span, and multiple reproductive events in life within a promiscuous mating system. As increased reproduction during early stages (i.e., typical strategy) can impose constraints on energy usage by females through a conflict in the need for growth and development between the mother and offspring (Pittet et al., 2017), the delay‐overshoot pattern in mean age‐specific fertility displayed by females may be a trade‐off‐induced adaptive life history decision (McNamara & Houston, 1996). Conversely, major hurricanes early in life may impose mortality pressure on affected females at young ages that could result in lower LRS. Yet, we found no evidence for hurricane‐induced mortality risk. The apparent low survival risk during adulthood showed by affected females suggests adversity‐induced age‐specific survival costs and differential fitness. That is, affected females surviving to adulthood (our focal subjects for reproductive metrics) may possess higher quality traits than those dying at immature ages. For instance, lower infant body mass, which represents a potential consequence of early‐life adversity, is associated with an increased risk of dying before maturation (Lee et al., 2020). In this way, variation in individual quality may mask expected trade‐offs (McLean et al., 2019). Although major hurricanes are known to have immediate effects on the mean annual fertility of these females, they have no effects on annual survival, a finding that motivated hypotheses about the potential optimization of life histories by means of ensuring survival on a bad year at the cost of reproduction (Morcillo et al., 2020). Finally, our analysis showed that experiencing a hurricane early in life slightly increased the chances of having an LRS equal to 0. This partly contributed to a difference of one offspring in median LRS between cohorts (~14% relative fitness difference). Yet, the magnitude of the hurricane effect in our model was small, and the overlap in the distribution of LRS between both female cohorts was significant. Our analysis supports instead that the combined increased fertility and maintenance of reproductive pace mainly allowed females experiencing a major hurricane to recover from a delayed reproductive debut.

### 4.2 Density‐induced early‐life adversity

Increasing density early in life was associated to a suppressed reproduction; however, these associations became weaker at intermediate densities of ~170–300 adult females. Nonlinear density dependence in fertility has been reported previously in this population who showed optimal annual mean fertility at intermediate densities (Hernández‐Pacheco & Steiner, 2017), highlighting the potential challenge of competing for mates at low densities and for resources at high densities. Density‐dependent delays in reproductive debut on a given year have also been observed in Cayo Santiago females (Bercovitch & Berard, 1993). Although Cayo Santiago is provisioned with food, feeding stations remained constant across the study period resulting in differential time to food access among individuals and thus resembling wild populations with relatively abundant resources. The latter is likely because the social structure of female rhesus macaques generates within‐group contest competition and among‐group competition (i.e., group‐level rank), and thus, food availability does not translate into stable food consumption by all individuals (Bercovitch & Berard, 1993; Sterck et al., 1997). Density dependence in fertility may also be supported through aggressive interactions between kin and nonkin (Judge & De Waal, 1997). Thus, high‐density early in life may expose females to both nutritional and psychosocial adversity.

Increasing density increased LRS among those females with the chance of reproducing. This may be driven by birth cohorts experiencing intermediate densities. It is important to mention that the cohort truncated data for the LRS analysis may have reduced negative density effects in our analysis. Increasing density also increased the chances of having an LRS equal to 0 by ~2%, suggesting induced adult mortality risk. Our analysis revealed that females experiencing increased density early in life also experienced an increase in hazard at every age passed the first week of life but these associations were significant during mainly immature ages (1 week to 3.5 years of age) and very old ages (>16.5 years of age). This is important for our study as it suggests that the observed reproductive compensation of females may be possible due to such reduced mortality pressure among (prime) adult ages (>3.5–16.5 years of age). That is, as long as an early‐life adversity affected female makes it into adulthood (surviving the vulnerable young ages), she may be able to contribute to population growth without being exposed to strong mortality risk. In this way, our study reveals that density regulates this population by acting on females at multiple live stages.

### 4.3 Implications to life history theory

Our study reveals an alternative life history strategy in this long‐lived species to that proposed by the PAR of accelerated reproduction following early‐life adversities. Female rhesus macaques experiencing early‐life ecological adversity suffered delays in reproduction but were able to compensate for it later in life. For long‐lived primates, life span is an important predictor of LRS (Blomquist, 2013; Robbins et al., 2011; Weibel et al., 2020). For Cayo Santiago rhesus macaques, LRS was strongly associated to life span. Per one unit increase in age at death, LRS increased by ~8%. Previous studies at Cayo Santiago also found that differences in reproductive timing are not associated with significant differences in LRS (Bercovitch & Berard, 1993). Thus, accelerated reproduction is not supported as an adaptive response to early‐life adversity in Cayo Santiago rhesus macaque females. Instead, our study suggests potential trade‐offs between survival and reproduction in which adversity‐affected females allocate more energy to growth or maintenance processes at younger adult ages to ensure future reproductive potential (Morcillo et al., 2020; Thompson, 2017).

Our study reflects previous findings in other long‐lived nonhuman primate populations in which accelerated reproduction does not result in a favored strategy among females experiencing nutritional and psychosocial sources of early‐life adversity (Weibel et al., 2020). Our analysis also provides new information on potential adaptive mechanisms following harsh conditions early in life, including natural disasters. Research on how extreme climatic events act as a source of early‐life ecological adversity is increasingly relevant as climate change is expected to increase both the prevalence and severity of major hurricane events (Knutson et al., 2020) with detrimental consequences on primate population viability (Ameca Y Juárez et al., 2015). As data from long‐term studies accumulate, new opportunities to contribute to the early‐life adversity and cohort effects frameworks become possible. Here, we provide further insight into factors that shape the reproductive life history of individuals within populations exposed to major natural disasters and reduced resources during developmental stages highlighting the need for studies addressing the demographic mechanisms for life history optimization following adverse conditions early in life.

## CONFLICT OF INTEREST

We declare no conflict of interest.

## AUTHOR CONTRIBUTIONS

**Logan Luevano:** Formal analysis (equal); Investigation (equal); Visualization (equal); Writing – original draft (lead); Writing – review & editing (equal). **Chris Sutherland:** Investigation (supporting); Methodology (supporting); Writing – review & editing (equal). **Stephanie J. Gonzalez:** Formal analysis (equal); Investigation (supporting); Visualization (equal); Writing – review & editing (supporting). **Raisa Hernández‐Pacheco:** Conceptualization (lead); Formal analysis (equal); Funding acquisition (lead); Investigation (equal); Methodology (lead); Project administration (lead); Resources (lead); Supervision (lead); Writing – original draft (supporting); Writing – review & editing (equal).

## Supporting information

Supplementary Material

## Data Availability

Data are available in Dryad (https://doi.org/10.5061/dryad.v9s4mw6xj).
