# Supplementary material for: Rhesus macaques compensate for reproductive delay following ecological adversity early in life
Source: Ecol Evol. 2022 Jan 12;12(1):e8456. doi: 10.1002/ece3.8456 (PMC8809442; doi:10.1002/ece3.8456)
Supplement: Supplementary file 1 — Supplementary Material [file ECE3-12-e8456-s001.docx]

**Electronic Supplemental Material**

Rhesus macaques compensate for reproductive delay following ecological adversity early in life

Logan Luevano^1^, Chris Sutherland^2^, Raisa Hernández-Pacheco^1^

^1^Department of Biological Sciences, California State University-Long Beach, 1250 Bellflower Blvd, Long Beach, CA 90840, ^2^The Center for Research into Ecological and Environmental Modeling, University of St. Andrews, St. Andrews, Fife, KY16 9AJ, Scotland

**Table S1**: Brant Test coefficients for the ordinal logistic regression model.

| Parameter | Coefficient | df | *p*-value |
| --- | --- | --- | --- |
| Global | 1.24 | 2 | 0.54 |
| $\beta_{nonhurricane}$ | 0.91 | 1 | 0.34 |
| $\beta_{density}$ | 0.23 | 1 | 0.63 |

| Model | Chi-square | df | p |
| --- | --- | --- | --- |
| density | 5.32 | 1 | 0.02 |
| global | 5.32 | 1 | 0.02 |

**Table S2**: Proportional hazard assumption violation check using the top model from Table S8.

Figure S3: The effect of density at birth on mortality varies over time. The black solid line is the time-varying hazard, and the black dashed lines are lower and upper limits of confidence interval (n = 4,236) of the density effect. Following this, a decision was made to cut time at 0.015 years (approaching 0), 3.5 years (point of inflection) and 16.5 years (point of inflection) resulting with 4 different time groups. The red line crossing the y-axis at 0 is the reference line for null effect.

**Table S4**: Proportional hazard assumption violation check for the time dependent coefficient (density at birth) cox proportional hazards regression model extension model from **Table S11**.

| Model | Chi-square | df | p |
| --- | --- | --- | --- |
| density: strata(time-group) | 6.79 | 4 | 0.15 |
| global | 6.79 | 4 | 0.15 |

**Table S5**: Model selection for generalized additive mixed models testing for variability in mean age-specific fertility of Cayo Santiago rhesus macaque females as a function of cohort type (hurricane, non-hurricane) and population density at birth. All models included individual ID as random intercepts.

| Model ~ | df | logLik | AICc | $\Delta AIC$ | $\Omega$ |
| --- | --- | --- | --- | --- | --- |
| cohort + s(age, by cohort) + s(density) | 9 | -8658.1 | 17334 | 0.0 | 0.90 |
| s(age, by cohort) + s(density) | 8 | -8661.4 | 17339 | 4.3 | 0.10 |
| Cohort + s(age) + s(density) | 7 | -8685.1 | 17384 | 49.9 | 0.00 |
| s(age) + s(density) | 6 | -8689.7 | 17391 | 57.0 | 0.00 |
| s(age, by cohort) | 6 | -8750.0 | 17512 | 177.7 | 0.00 |
| Cohort + s(age, by cohort) | 7 | -8750.0 | 17514 | 179.7 | 0.00 |
| s(age) | 4 | -8769.0 | 17546 | 211.5 | 0.00 |
| Cohort + s(age) | 5 | -8768.7 | 17548 | 213.1 | 0.00 |
| null | 2 | -9462.3 | 18929 | 1594.3 | 0.00 |

**Note**: cohort = hurricane, non-hurricane; s() corresponds to smooth terms, s(by) correspond to a factor smooth interaction.

**Figure S6**: Partial effects of the top GAMM model in **Table S5** which includes effects of age across cohort type, density, and cohort on age-specific fertility.

|  |  |
| --- | --- |
|  |  |

**Table S7**: Model selection for ordinal logistic regression models testing for variability in mean age at reproductive debut of Cayo Santiago rhesus macaque females as a function of cohort type (hurricane, non-hurricane) and population density at birth.

| Model ~ | df | logLik | AICc | $\Delta AIC$ | $\Omega$ |
| --- | --- | --- | --- | --- | --- |
| cohort + density | 4 | -1393.1 | 2794.3 | 0.0 | 1.0 |
| cohort | 3 | -1413.1 | 2832.2 | 37.3 | 0.0 |
| density | 3 | -1416.8 | 2839.7 | 45.4 | 0.0 |
| null (intercept only) | 2 | -1437.2 | 2878.4 | 84.3 | 0.0 |

**Table S8**: Coefficients and intercepts of the top ordinal logistic regression model in **Table S7** which includes effects of age and density on the age at reproductive debut.

|  | Value | SE |
| --- | --- | --- |
| *Coefficient* |  |  |
| cohort | -0.925 | 0.138 |
| density | 0.003 | 0.001 |
| *Intercepts* |  |  |
| 3 \| 4 | -0.455 | 0.200 |
| 4 \| 5 | 2.884 | 0.215 |

**Table S9**: Model selection for generalized additive mixed models testing for variability in the probability of birth skipping of Cayo Santiago rhesus macaque females as a function of cohort type (hurricane, non-hurricane) and population density at birth. All models included individual ID as random intercepts.

| Model ~ | df | logLik | AICc | $\Delta AIC$ | $\Omega$ |
| --- | --- | --- | --- | --- | --- |
| s(age) + s(density) | 6 | -6382.5 | 12777.0 | 0.0 | 0.66 |
| cohort + s(age) + s(density) | 7 | -6382.2 | 12778.5 | 1.5 | 0.32 |
| s(age, by cohort) + s(density) | 8 | -6384.3 | 12784.6 | 7.6 | 0.02 |
| cohort + s(age, by cohort) + s(density) | 9 | -6383.9 | 12785.9 | 8.9 | 0.01 |
| cohort + s(age) | 5 | -6467.1 | 12944.3 | 167.2 | 0.00 |
| s(age) | 4 | -6468.4 | 12944.8 | 167.8 | 0.00 |
| cohort + s(age, by cohort) | 7 | -6473.0 | 12960.0 | 183.0 | 0.00 |
| s(age, by cohort) | 6 | -6474.4 | 12960.8 | 183.7 | 0.00 |
| null (intercept only) | 2 | -6699.0 | 13402.1 | 625.1 | 0.00 |

**Note**: cohort = hurricane, non-hurricane; s() corresponds to smooth terms, s(by) correspond to a factor smooth interaction.

**Table S10:** Model selection for zero-inflated negative binomial distribution due to overdispersion.

| Model ~ | df | logLik | AICc | $\Delta$AIC | $\Omega$ |
| --- | --- | --- | --- | --- | --- |
| density + exit \| cohort + density + exit | 8 | -816.8 | 1650.0 | 0.0 | 0.65 |
| density + exit \| density + exit | 7 | -818.5 | 1651.3 | 1.2 | 0.35 |
| density + exit \| exit | 6 | -824.4 | 1661.1 | 11.0 | 0.00 |
| density + exit \| 1 | 5 | -909.3 | 1828.8 | 178.8 | 0.00 |

**Table S11**: Model selection for Cox proportional hazards regression model testing for time to death of Cayo Santiago rhesus macaque females as a function of early life ecological adversities; cohort type (hurricane, non-hurricane) and population density at birth.

| Model ~ | df | logLik | AIC_C_ | ΔAIC_c_ | Ω |
| --- | --- | --- | --- | --- | --- |
| density | 1 | -11066.8 | 22135.6 | 0.00 | 0.64 |
| cohort + density | 2 | -11066.8 | 22137.6 | 1.95 | 0.24 |
| null (intercept only) | 0 | -11069.8 | 22139.6 | 4.03 | 0.09 |
| cohort | 1 | -11069.8 | 22141.6 | 5.99 | 0.03 |

**Table S12**: Coefficients for the time varying cox proportional hazards regression model.

|  | Coefficient estimate | | 95% CI | |
| --- | --- | --- | --- | --- |
|  | *e^β^* | SE | Lower | Upper |
| Density: < 0.02 years | 0.994 | 0.0011 | **0.992** | **0.996** |
| Density: 0.02 – 3.5 years | 1.002 | 0.0005 | **1.001** | **1.003** |
| Density: 3.5 – 16.5 years | 1.000 | 0.0006 | 0.999 | 1.001 |
| Density: > 16.5 years | 1.002 | 0.0010 | **1.000** | **1.004** |

Bolded 95% CI do not overlap with 0 (p < 0.05).
